# Teacher Support, Academic Self-Efficacy, Student Engagement, and Academic Achievement in Emergency Online Learning

**DOI:** 10.3390/bs13090704

**Published:** 2023-08-24

**Authors:** Liang Huang, Dongsheng Wang

**Affiliations:** 1 Department of Public Administration, Southeast University, Nanjing 211189, China; 2 Faculty of Education, Northwest Normal University, Lanzhou 730070, China

**Keywords:** teacher support, academic self-efficacy, student engagement, academic achievement

## Abstract

With a sample of 651 university students experiencing emergency online learning during COVID-19, this study constructed a structural equation modelling to examine the effects of teacher support on students’ academic achievement, with a particular focus on the mediating roles of academic self-efficacy and student engagement. The results show that teacher support had significant total influences on university students’ academic achievement. Furthermore, academic self-efficacy and student engagement, respectively, mediated the effects of teacher support on students’ academic achievement. In addition, academic self-efficacy and student engagement sequentially mediated the effects of teacher support on students’ academic achievement. Research implications are also discussed.

## 1. Introduction

Since the massive outbreak of the Coronavirus Disease 2019 (COVID-19), lockdown and social distancing measures have been implemented in countries around the world as a way to curb the spread of the virus [1]. As an emergency response, universities were forced to close and move to online platforms to continue educational activities remotely. While the implementation of emergency online learning ensured educational continuity in this unprecedented situation, it also exposed students to significant routine disruptions and huge learning shocks, negatively impacting their engagement and academic achievement [2].

Against this backdrop, teacher support, or teacher’s support of students enabling them to feel that they are valued and supported and that their teachers can be relied on for assistance in learning [3], has been highlighted by researchers as increasingly important in sustaining students’ engagement and achievement [4]. Indeed, teacher support represents the full potential for students to address various obstacles to emergency online learning, including unfamiliar technical environments, confusing instructional strategies, non-structured learning materials, and intermittent distractions at home [5]. This is particularly so during COVID-19, as students lacked access to school services and missed out on the valuable in-person interactions that they had previously benefited from [6]. Despite the need for enhanced teacher support to help students foster academic efficacy in handling learning requirements and maintain engagement to achieve academic goals [7,8], only few studies have examined the effects of teacher support on students’ academic achievement in emergency online learning during the pandemic [9], and fewer still go further to explore the mechanisms underlying these effects, impeding development of target measures to better support students in meeting their learning needs.

Based on the discussions above, we in this study attempted to examine the effects of teacher support on university students’ academic achievement in emergency online learning during COVID-19, with a particular interest in the mediating roles of academic self-efficacy and student engagement. These two variables were examined as crucial mediators in that research has highlighted teacher support as a proximal social determinant of students’ academic self-efficacy and engagement in course learning [10,11], both of which contribute to the multilayered personal factors that interact to affect students’ academic achievement [12,13]. This is exactly the case in the context of emergency online learning, given the irreplaceable role that teacher support plays in fostering students’ beliefs in overcoming exceptional challenges and sustaining their engagement in academic activities [6,14]. Since emergency online learning will continue to be an essential part of the educational landscape in the post-COVID era [15], the research findings can provide useful implications for university managers and teachers to adapt and improve online teaching practices in ways of fostering student engagement and promoting academic achievement.

## 2. Literature Review

### 2.1. Teacher Support and Academic Achievement in Emergency Online Learning during COVID-19

In the existing literature, teacher support, which denotes the beneficial experiences of supportive teacher–student relations, has been identified as particularly influential in helping students overcome learning frustrations, promoting meaningful engagement in coursework, and sustaining students’ academic progress [3,16]. In practice, teachers can support students in multiple ways, such as displaying an interest in their academic progress, making efforts to find out the difficulties they face, providing prompt feedback and encouragement, and offering extra assistance whenever required [17]. Research has consistently shown positive effects of teacher support on academic achievement [7,18]. Specifically, students who feel supported by teachers are more likely to exhibit positive academic beliefs, resilience, and adaptive goals toward learning activities and tend to invest more efforts in making progress in their course work [19]. In fact, teacher support plays a vital role in shaping an effective and facilitative learning environment that stimulates students’ motivation and engagement in course learning [20]. The sudden transition into emergency online learning during COVID-19 gave prominence to the role of teacher support in helping students navigate the unprecedented disruptions and learning challenges, and sustain their academic achievement [4]. In this unique context, students can greatly benefit from the support provided by teachers in their online learning, as it helps to offset losses in achievement due to the closure of university services and the lack of in-person interactions [6]. Hence, it is reasonable to hypothesize that:

**H1.**  *Teacher support had significant and positive effects on university students’ academic achievement in emergency online learning during COVID-19*.

### 2.2. Mediating Role of Academic Self-Efficacy

Academic self-efficacy refers to individuals’ beliefs in their competence to successfully accomplish a set of course learning tasks as expected [21]. As an internal resource that drives motivation, academic self-efficacy plays a pivotal role in stimulating confidence and persistence in the face of obstacles and in promoting goal-orientated planning and self-regulated actions in pursuit of desired academic outcomes [22,23]. In the existing literature, evidence of empirical research has consistently identified academic self-efficacy as a robust predictor of students’ academic achievement, as measured through various indicators such as GPA, dropouts, and self-reported academic performance [24,25]. This can be especially true in the context of emergency online learning during COVID-19 as students are found to heavily rely on their personal beliefs as an internal strength to offset pandemic-related impacts and the concomitant learning loss [14].

The research also indicates that receiving proximal support from teachers is closely linked to a boost in academic self-efficacy [26]. As informed by the sources of self-efficacy theory [22], teachers who demonstrate constructive feedback regarding students’ progress and encourage them to be confident in overcoming obstacles can offer social persuasion that promotes their academic self-efficacy [10]. Teachers may also help students accumulate vicarious experiences through introducing peer examples and effective coping strategies in course learning to enhance academic self-efficacy and academic development [27]. As evidenced, an increase in academic self-efficacy would in turn lead to an improvement in students’ academic achievement [8,28]. Considering the particular importance of teacher support in fostering students’ academic self-efficacy to address the exceptional challenges and retain academic progress during the COVID-19 emergency online learning period [29,30], it can be hypothesized that:

**H2.**  *Academic self-efficacy significantly mediated the effects of teacher support on university students’ academic achievement in emergency online learning during COVID-19*.

### 2.3. Mediating Role of Student Engagement

Student engagement refers to the time and effort students dedicate to participating in course-related learning activities and exercises [31]. A synthesis of literature indicates a multifaceted construct featuring agentic, behavioral, emotional, and cognitive dimensions of engagement with respect to effective learning [32]. In course learning contexts, engaged students not only work hard, experience enjoyment, and utilize advanced learning strategies when faced with tasks, but also proactively share insights and perspectives with teachers to contribute to their learning process [33]. Research has elucidated the salient role of student engagement in both physical classroom and online settings because it is recognized in its own right as a relevant indicator of quality learning and as a significant predictor of academic success [34,35]. Globally, the severe disruptions and learning shocks that students experienced during the COVID-19 emergency online learning period have had negative impacts on student engagement, leading to a significant loss in their academic achievement [36].

Research has also highlighted the importance of teacher support as a proximal social influence in shaping overall student engagement [11]. Empirical evidence has demonstrated that when teachers deliver support in course instruction, such as showing care and encouragement, offering scaffolding feedback and guiding clues, and providing personalized learning support, students’ engagement in learning and academic achievement can be significantly promoted [37]. This is especially true during the COVID-19 pandemic, as students have been suffering a significant absence of a conducive environment in relation to meaningful engagement in learning [6]. Given the salient role of teacher support as an integral aspect of social environment in mitigating the impacts of academic disengagement and diminished course experiences [38], researchers have urgently called for support from teachers to aid students in overcoming online learning challenges, so as to sustain their engagement and academic progress [39]. In this regard, it can be proposed that:

**H3.**  *Student engagement significantly mediated the effects of teacher support on university students’ academic achievement in emergency online learning during COVID-19*.

### 2.4. Sequential Mediation of Academic Self-Efficacy and Student Engagement

Academic self-efficacy represents a most influential psychological determinant of the academic engagement of university students [40]. Evidence has shown that students who possess a strong sense of academic efficacy are inclined to be actively engaged in course learning and take charge of their learning activities, while those with low levels of academic self-efficacy tend to disengage and perform poorly [41]. In fact, academic self-efficacy has been identified as particularly important in counterbalancing the pandemic-induced disruptions and in sustaining student engagement and academic progress during COVID-19 [42]. Research has also shown that teacher support as a proximal social factor interacts with student-based academic self-efficacy to enable students’ optimal academic functioning and maintain their engagement in learning [12,26]. For instance, Liu, Du and Lu [19] found that self-efficacy significantly mediated the effects of teacher support on university students’ engagement in EFL learning. Gutiérrez and Tomás [43] further revealed that teachers’ autonomy support had effects on university students’ academic success through exerting impacts on efficacy and engagement. Although no study, to our knowledge, has explored whether teacher support influences students’ academic achievement in emergency online learning via the sequential mechanisms of academic self-efficacy and student engagement, the empirical literature, as reviewed, regarding the influences of teacher support on academic self-efficacy [40,41], as well as the interactions of teacher support and academic self-efficacy in relation to student engagement and academic achievement [19,43], have led us to propose the following hypothesis:

**H4.**  *Teacher support influenced students’ academic achievement through the sequential mediation of academic self-efficacy and student engagement in emergency online learning during COVID-19*.

## 3. Methods

### 3.1. Data and Sample

University students experiencing COVID-19 emergency online learning were selected as the research sample in this study. Specifically, a combination of convenience sampling and snowball sampling procedures was used to select potential participants in 2020. Specifically, a 20 min anonymous digital questionnaire generated by the online questionnaire platform of Wenjuanxing (https://www.wjx.cn/ (accessed on 15 July 2020) was administered to available university staff, who then distributed it to available students. A total of 651 university students (30.1% males) who were enrolled in full-time undergraduate courses were investigated to obtain valid responses regarding their emergency online learning experiences during COVID-19. Of the participants, there were 226 (34.7%) in grade one, 246 (37.8%) in grade two, and 179 (27.5%) in grade three or higher.

Ethical guidelines were observed throughout the digital questionnaire investigation. On the one hand, research ethical approval was obtained from the Research Ethics Committee of the corresponding author’s affiliated university department before participant selection and data collection. On the other hand, all participants gave their consent to participate in the anonymous questionnaire investigation after being fully informed of the research purpose as well as the right to quit for any reason at any time.

### 3.2. Measures

#### 3.2.1. Teacher Support

Teacher support was measured with 4 items adapted from the Course Experience Questionnaire [17]. Students were asked to report their perceptions of support from teachers during the emergency online learning period on a 6-item Likert scale that ranges from 1 (strongly disagree) to 6 (strongly agree), and one example was “Teachers make a real effort to understand difficulties students may be having with their work”. The calculated Cronbach’s α (0.805) for the teacher support scale showed good reliability. CFA results indicated that the teacher support scale had a good construct validity (χ^2^ = 2.723, df = 2, RMSEA = 0.024, CFI = 0.999, TLI = 0.997) with high factor loadings ranging from 0.620 to 0.770.

#### 3.2.2. Academic Self-Efficacy

University students’ academic self-efficacy was measured with 4 items adapted from Parker [44]. Students were asked to rate the beliefs in their capabilities of carrying out a set of broad academic tasks (e.g., “Examining a persistent course learning problem to find a solution”) during emergency online learning period on a Likert scale that ranges from 1 (strongly disagree) to 6 (strongly agree). The calculated Cronbach’s α (0.833) for the academic self-efficacy scale showed good reliability. CFA results indicated that the academic self-efficacy scale had a good construct validity (χ^2^ = 3.262, df = 2, RMSEA = 0.031, CFI = 0.999, TLI = 0.996) with high factor loadings ranging from 0.669 to 0.799.

#### 3.2.3. Student Engagement

University students’ engagement in emergency online learning was measured with the Student Engagement Scale [32]. The 22-item scale contains four dimensions: (1) agentic engagement (5 items, e.g., “I offer suggestions about how to make the online class better”); (2) behavioral engagement, (5 items, e.g., “I pay attention in online class”); (3) emotional engagement (4 items, e.g., “I enjoy learning new things in online class”); (4) cognitive engagement (8 items, e.g., “When I study, I try to connect what I am learning with my own experiences”). Students were asked to rate the items on a six-point Likert scale that ranges from 1 (strongly disagree) to 6 (strongly agree). The calculated Cronbach’s α (0.955) for the overall student engagement scale showed good reliability, as did the subscales of agentic (0.910), behavioral (0.920), emotional (0.897), and cognitive engagement (0.911). First-order CFA showed good construct validity for the student engagement scale (χ^2^ = 899.191, df = 203, RMSEA = 0.073, CFI = 0.937, TLI = 0.928). To demonstrate students’ overall online engagement, a second-order CFA model was constructed, validating the overall construct (χ^2^ = 902.363, df = 205, RMSEA = 0.072, CFI = 0.937, TLI = 0.929) with high factor loadings ranging from 0.697 to 0.968.

#### 3.2.4. Academic Achievement

University students’ academic achievement in emergency online learning was measured through a self-reported scale as indicated by Chen [38]. Students were asked to rate on the single-item academic achievement scale through answering the question: “Overall, how do you evaluate your academic achievement in emergency online learning during COVID-19?” The scale ranges from 1 to 6, with 1 indicating the worst academic achievement and 6 the best.

### 3.3. Data Analysis

SPSS 24.0 and Mplus 7.4 software were employed to analyze data. First, correlation analysis was conducted by SPSS to examine correlations. Then, structural equation modeling (SEM) was constructed by Mplus to calculate the relationships among focus variables and conduct mediation analysis. University students’ gender (1 = male and 0 = female) and grade were chosen as control variables in this study. The use of SEM allowed us to simultaneously address measurement errors and calculate structural parameters [45]. In addition, the bias-corrected bootstrap method with 2000 resamples was employed to estimate the 95% confidence intervals for mediating effects [45]. According to Hu and Bentler [46], the SEM model was identified as acceptable if: root-mean-square error of approximation (RMSEA) < 0.08; Tucker–Lewis index (TLI) > 0.90; and comparative fit index (CFI) > 0.90.

Furthermore, SPSS software was used to examine potential common method bias through Harman’s single factor test. Since less than 50% (43.23%) of total variance was explained by one common factor, the common method bias did not affect the data and hence the results [47].

## 4. Results

### 4.1. Correlation Analysis

The results of correlation analysis are shown in Table 1. As expected, university students’ academic achievement was significantly and positively correlated with student engagement (γ = 0.413, *p* < 0.001), academic self-efficacy (γ = 0.314, *p* < 0.001), and teacher support (γ = 0.196, *p* < 0.001). Furthermore, student engagement was significantly and positively correlated with academic self-efficacy (γ = 0.539, *p* < 0.001) and teacher support (γ = 0.548, *p* < 0.001). In addition, academic self-efficacy was significantly and positively correlated with teacher support (γ = 0.275, *p* < 0.001).

### 4.2. Structural Equation Modeling (SEM)

**Figure 1:**
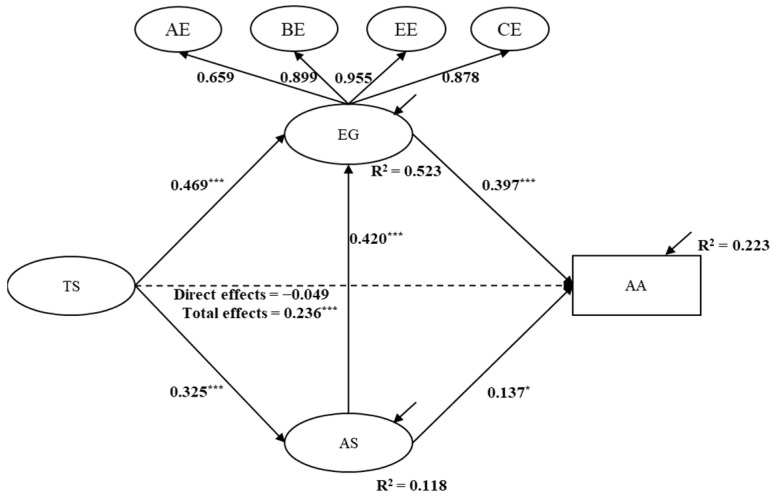
The SEM results. Note: Standardized coefficients are reported. TS = teacher support, AS = academic self-efficacy, EG = student engagement, AA = academic achievement. * *p* < 0.05, *** *p* < 0.001.

SEM analysis was conducted to calculate the relationships among focus variables with gender and grade being controlled and to conduct mediation analysis. The SEM results are displayed in Figure 1. In total, the model explained 22.3%, 52.3%, and 11.8% of the variance in academic achievement, student engagement, and academic self-efficacy, respectively. Furthermore, the model fit indices generated were: χ^2^ = 1542.94, df = 481; RMSEA = 0.058; CFI = 0.923; and TLI = 0.915, indicating a good SEM model fit.

As Figure 1 illustrates, teacher support had significant total influences on academic achievement (β = 0.236, *p* < 0.001) without the inclusion of the mediating variables of academic self-efficacy and student engagement, thus supporting the idea that teacher support had significant and positive effects on university students’ academic achievement in emergency online learning during COVID-19 (H1).

Teacher support did not have significant and direct influences on students’ academic achievement (β = −0.049, *p* > 0.05). Instead, teacher support had significant and positive influences on both academic self-efficacy (β = 0.325, *p* < 0.001) and student engagement (β = 0.469, *p* < 0.001). Furthermore, both academic self-efficacy (β = 0.137, *p* < 0.05) and student engagement (β = 0.397, *p* < 0.001) had significant and positive influences on academic achievement. Moreover, academic self-efficacy had significant and positive influences on student engagement (β = 0.420, *p* < 0.001).

The results of mediation analysis in Table 2 show that academic self-efficacy significantly mediated the effects of teacher support on academic achievement (β = 0.044, *p* < 0.05, 95% CIs: 0.011 to 0.093), thus supporting H2. In addition, student engagement significantly mediated the effects of teacher support on academic achievement (β = 0.186, *p* < 0.001, 95% CIs: 0.121 to 0.269), thereby supporting H3. In addition, academic self-efficacy and student engagement significantly and sequentially mediated the effects of teacher support on academic achievement (β = 0.054, *p* < 0.001, 95% CIs: 0.027 to 0.093). Therefore, H4 was supported.

## 5. Discussion

The emergency online learning implemented during COVID-19 has caused significant disruptions to university students’ learning engagement and academic achievement [2]. The exceptionally challenging situation has emphasized the urgent need for research into the important role of teacher support in bolstering student engagement and sustaining academic achievement [8,29]. However, empirical evidence remains scarce regarding whether and how teacher support impacted university students’ academic achievement. To fill the research gaps and to facilitate the development of effective intervention measures, this study examined the effects of teacher support on students’ academic achievement in emergency online learning during COVID-19, with a particular focus on the mediating roles of academic self-efficacy and student engagement.

In this study, teacher support was found to have significant total influences on student’s academic achievement in emergency online learning during COVID-19. Consistent with previous studies showing the significantly importance of teacher support in sustaining students’ motivation, perseverance, and engagement, and promoting students’ learning quality and academic achievement [19,20], the results corroborate that supportive teachers who value students’ self-regulated efforts, care for students’ academic progress, and offer extra guidance and assistance for students’ problem-solving assume a crucial role in fostering academic achievement [7,17]. In fact, researchers have identified a multitude of learning challenges that students face during the COVID-19 emergency online learning period, underscoring the need for teachers to provide support to help students overcome the severe disruptions of learning routines and maintain academic achievement [9,29].

Furthermore, this study reveals that academic self-efficacy significantly mediated the effects of teacher support on university students’ academic achievement. On the one hand, the results confirm that academic self-efficacy as a critical internal driving force plays a pivotal role in soliciting adaptive coping, boosting academic confidence, and sustaining academic achievement in the face of significant learning obstacles [22]. The results add credence to the claim of Wong and Yuen [14] that academic self-efficacy can be particularly relevant as it helps university students strengthen academic resilience to counterbalance the shock of emergency online learning during COVID-19. On the other hand, the results substantiate the significant and positive effects of teacher support as an exogenous proximal factor on students’ academic self-efficacy [26], which can be attributed to the fact that teachers’ instrumental, emotional, and academic support in course learning produced positive social persuasion and vicarious experiences that facilitate academic self-efficacy [10,27]. The findings further contribute to the literature through unravelling the role of academic self-efficacy in connecting teacher support and students’ academic achievement in emergency online learning during COVID-19. That is, when faced with unprecedented learning disruptions, students receiving various support from teachers can enhance their confidence in addressing challenges and stay focused on their academic progress [29,30].

In addition, this study confirms that student engagement significantly mediated the influences of teacher support on university students’ academic achievement. The results, on the one hand, reflect the empirical relevance of students’ agentic, behavioral, emotional, and cognitive engagement to learning achievement and academic progress [34,39]. The results also align with empirical research that links the significant decline in academic outcomes during COVID-19 emergency online learning to reduced student engagement [36]. Additionally, the results reveal the significant and positive effects of teacher support on student engagement, which was in line with previous studies elucidating teacher support as a proximal social determinant of overall student engagement [37,38]. The research findings provide further evidence regarding the significant mediating role of student engagement in connecting teacher support and academic achievement. In this sense, student engagement was identified as a critical mechanism that explains the variance in student academic achievement attributable to teacher support [7]. This is especially true in the context of emergency online learning during COVID-19, as students heavily rely on the support of teachers to sustain engagement and achievement [6]. The findings thus contribute to the literature by highlighting that the effectiveness of teacher support lies in its ability to assist students in overcoming learning obstacles and sustaining full engagement [48].

Moreover, this study reveals that teacher support exerted influences on academic achievement through the sequential mediation of academic self-efficacy and student engagement. The results, on the one hand, verified the importance of academic self-efficacy as a key psychological factor in influencing student engagement and academic performance [40,49], especially when university students were in the face of unprecedented learning challenges triggered by the COVID-19 pandemic [42]. On the other hand, the results elucidated that the proximal social resource of teacher support interacted with the internal psychological resource of academic self-efficacy to influence university students’ engagement and academic achievement [12,26]. In this sense, we have successfully filled a gap by unravelling, for the first time, a sequential mediating mechanism that sheds light on how teacher support affects the academic achievement of university students in emergency online learning during COVID-19.

## 6. Implications

Some practical implications can be generated based on the research results. First, this study found that teacher support had significant total effects on university students’ academic achievement in emergency online learning during COVID-19. The results suggest that teacher support can be critically important in helping students address learning challenges and sustain academic achievement in such an emergency situation. To this end, teachers may adopt a variety of measures, including providing instrumental support (e.g., using scaffolding questions and successive hints in online courses), delivering emotional care in interaction with students (e.g., maintaining regular communications and active listening), offering academic guidance and feedback to combat online distractions (e.g., modelling good online learning behaviors and time management skills), and empowering students to take control of their learning (e.g., setting guidelines for online participation and offline self-evaluation) [29,50]. By doing so, teachers can create a favorable learning environment that offsets the learning crisis and facilitates student academic progress [38].

Furthermore, this study found that academic self-efficacy and student engagement respectively mediated the effects of teacher support on university students’ academic achievement. This implies that effective teacher support should prioritize enhancing students’ academic self-efficacy and promoting their engagement in course learning within the context of emergency online learning. In practice, teachers may make efforts to enhance students’ self-confidence through demonstrating positive social persuasion (e.g., giving positive feedback and appraisal via email or digital apps) and helping them accumulate vicarious experiences (e.g., arranging virtual peer mentoring and online discussion forums) related to mastering learning tasks [22,51]. In addition, teachers may incorporate interactive and engaging contents, tools, and strategies in online teaching to sustain academic engagement and achievement [6].

Moreover, this study found that teacher support influenced university students’ academic achievement through the sequential mediating roles of academic self-efficacy and student engagement. Hence, when developing teaching strategies to promote students’ academic performance and achievement in similar situations, university teachers should simultaneously attend to the academic self-efficacy of students and their agentic, behavioral, emotional, and cognitive engagement. To better address it, universities should establish specifically tailored training programs to help teachers effectively enhance the efficacy beliefs and engagement of students struggling with the transition to emergency online learning.

## 7. Conclusions and Limitations

The research advances empirical understanding as to whether and how teacher support exerted influences on university students’ academic achievement in emergency online learning during COVID-19. In line with previous studies indicating that teacher support plays a critically important role in helping students address unprecedented disruptions and sustaining academic achievement [9,29], this study found significant and positive effects of teacher support on university students’ academic achievement. In particular, this study contributed to the literature by exploring the mediating roles of academic self-efficacy and student engagement regarding these effects.

Despite the research significance, there remain several limitations. First, a combination of convenience sampling and snowball sampling was employed to select participants, which may limit the representativeness of the research findings. Future studies that use more representative data are expected to validate the results. Second, the research was done with a cross-sectional research design, which cannot warrant causal references. Future studies may employ a longitudinal research design to examine causalities. Third, students’ academic achievement was measured using a self-reported method, which may introduce social desirability bias that can affect the interpretation of the results. Future studies may use students’ GPA as a proxy measure to validate the research findings. Finally, teacher support was examined as a critical proximal influence shaping student engagement and academic achievement. Future studies may explore the interaction between teacher support and other external environmental factors, such as socioeconomic status, in influencing students’ academic achievement in emergency online learning situations.

## Figures and Tables

**Table 1:** Results of correlation analysis.

	1	2	3	4
1. Academic achievement	1			
2. Student engagement	0.413 ***	1		
3. Academic self-efficacy	0.314 ***	0.539 ***	1	
4. Teacher support	0.196 ***	0.548 ***	0.275 ***	1
Mean	3.647	4.097	4.215	2.811
SD	0.988	0.746	0.778	0.519

Note: Standardized coefficients are reported. *** *p* < 0.001.

**Table 2:** Results of mediation analysis.

	Β	S.E.	95% Confidence Intervals
Teacher support → academic achievement (Direct effects)	−0.049	0.068	[−0.184, 0.082]
Teacher support → academic self-efficacy → academic achievement	0.044 *	0.021	[0.011, 0.093]
Teacher support → student engagement → academic achievement	0.186 ***	0.037	[0.121, 0.269]
Teacher support → academic self-efficacy → student engagement → academic achievement	0.054 ***	0.017	[0.027, 0.093]

Note: Standardized coefficients are reported. * *p* < 0.05, *** *p* < 0.001.

## Data Availability

The data presented in this study are available on request from the corresponding author.
